# Synergistic effect of Al_2_O_3_-decorated reduced graphene oxide on microstructure and mechanical properties of 6061 aluminium alloy

**DOI:** 10.1038/s41598-024-67004-x

**Published:** 2024-07-13

**Authors:** Hongding Wang, Haitao Zheng, Mingshuai Hu, Zhonglei Ma, Hong Liu

**Affiliations:** 1 School of Mechanical Engineering, Lanzhou Jiaotong University, Lanzhou, 730070 People’s Republic of China; 2 Urumqi West Depot, China Railway Urumqi Group Co.Ltd, Urumqi, 830023 People’s Republic of China

**Keywords:** Al_2_O_3_/reduced graphene oxide, Aluminium matrix composites, Strength, Synergistic effect, Mechanical engineering, Structural materials, Metals and alloys

## Abstract

In this study, Al6061 alloy matrix composites reinforced Al_2_O_3_-decorated reduced graphene oxide (Al_2_O_3_/RGO) with 0.1, 0.3 and 0.5 weight present (wt%) were successfully fabricated using high energy ball milling and hot extrusion techniques. The microstructures of these Al_2_O_3_/RGO/Al6061 aluminum matrix composites (Al MMCs) were characterized. The results showed that Al_2_O_3_/RGO were uniformly distributed within the Al6061 matrix and tightly bonded to the matrix. Al_2_O_3_ encapsulation on RGO surface would prevent the formation of Al_4_C_3_ brittle phase in matrix, ensuring that there was no reaction between the reinforcement and the matrix Al6061. Tensile strength and Vickers hardness tests demonstrated that the mechanical properties of Al MMCs significantly increased with addition of Al_2_O_3_/RGOs. Remarkably, Al MMCs with 0.1 wt% reinforcement showed tensile yield and tensile strengths of 270 MPa and 286 MPa, respectively, which were 49% and 43% higher than those of pure Al6061 prepared using the same process. Furthermore, the 0.1 wt% Al_2_O_3_/RGO composite also showed the best plastic deformation capability in considering of the strength.

## Introduction

Metal matrix composites have good physical and mechanical qualities, making them ideal for manufacturing lightweight structural components with high specific strength and specific modulus, which are widely employed in aerospace, aviation, automotive industries, and other disciplines^1,2^. Aluminum Metal Matrix Composite (Al MMC) are one of the most extensively utilized within this class of materials. They exhibit good ductility, durability, high specific strength and modulus, low coefficient of thermal expansion, excellent high-temperature properties, as well as good fatigue resistance and wear resistance^3^. Combined with their ease of processing, engineering reliability, and affordability, Al MMCs present favorable conditions for their utilization in engineering applications^4^. The reinforcement of Al MMCs primarily involves the use of oxide ceramic particles^5–12^, carbides^13–20^, nitrides^21^, and borides^22–24^, employing both non-in-situ and in-situ methods.

In recent year, graphene, renowned for its remarkable properties such as high strength, excellent thermal conductivity, and an extremely low coefficient of thermal expansion^25,26^, is considered an exceptional reinforcement in composite materials. Researchers have developed various methodologies to demonstrate that the incorporation of graphene enhances the mechanical, thermal, and tribological properties of metallic materials^27,28^. However, in the production of graphene/aluminum composites, graphene is prone to the easy breakage of the six-membered ring structure, poor wettability at the aluminum interface, and agglomeration^29^. Additionally, graphene readily reacts with the aluminum element in the aluminum alloy, leading to the formation of the brittle Al_4_C_3_ phase, which results in a decline in the performance of the Al MMCs^30,31^.

Therefore, in this study, Al_2_O_3_/reduced graphene oxide (RGO) nanoparticles (Al_2_O_3_/RGO) with a specific layered structure were synthesized by loading Al_2_O_3_ particles onto RGO through a hydrothermal process. The presence of Al_2_O_3_ could inhibit the reaction between RGO and Al element to form Al_4_C_3_ brittle phase. Subsequently, the Al MMCs were manufactured using a powder metallurgy method, incorporating them into the matrix Al6061 as the reinforcing phase. The aim is to investigate the impact of the Al_2_O_3_/RGO’s synergistic reinforcement on the mechanical properties of the matrix Al6061.

## Materials and experiments

### Material

**Figure 1:**
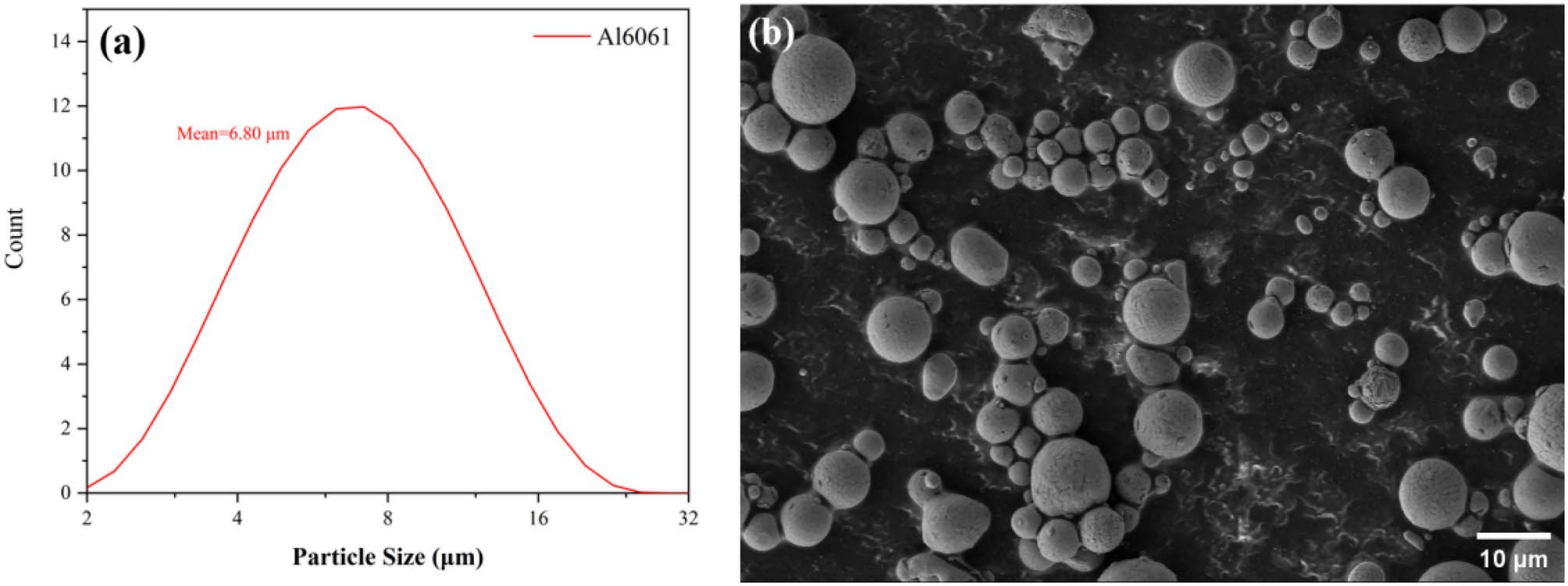
Particle diameter distribution (**a**) and the morphology (**b**) of Al6061 powder.

Al6061 alloy has outstanding mechanical characteristics because it is a precipitation solidified aluminum alloy composed primarily of magnesium and silicon. Therefore, in this investigation, Al6061 alloy powder was used as the foundation material. The content of other metallic elements in Al6061 is detailed in Table 1. The particle size distribution of Al6061 powder was analyzed using the Mastersizer 3000 laser diffraction particle size analyzer, as illustrated in Fig. 1a. The average diameter of Al6061 particles was determined to be 6.8 µm, and the morphology is shown in Fig. 1b.

**Table 1:** Chemical composition of Al6061 alloy (wt%).

Al	Si	Mg	Fe	Cu	Mn	Gr	Zn	Ti	O
97.47	0.63	1.08	0.17	0.32	0.052	0.18	0.032	0.02	0.16

### Synthesis of RGO and Al_2_O_3_/RGO composites

**Figure 2:**
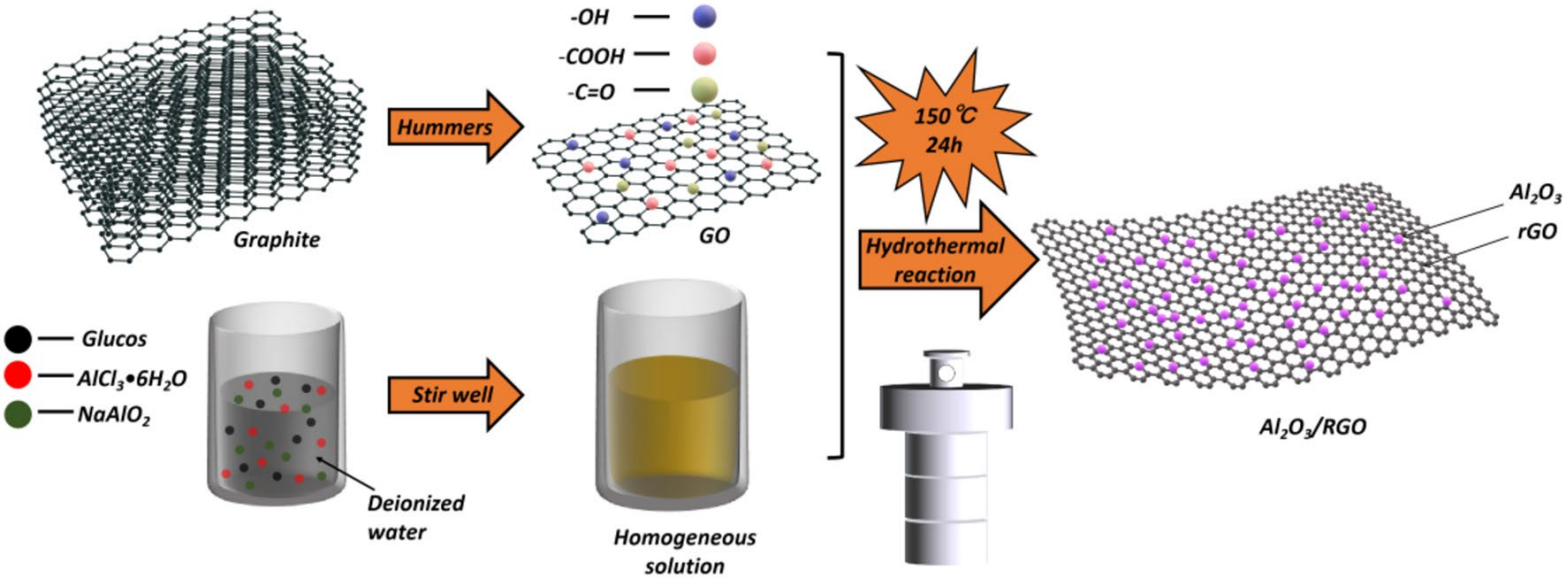
Synthesis process of Al_2_O_3_/RGO.

In this study, RGO flakes were firstly synthesized from flake graphite using a modified hummers method^32^. To achieve a well-dispersed graphene oxide (GO) solution, 0.54 g of GO was initially dispersed in 30 ml of deionized water, stirred with a magnetic stirrer for 2 h, and subsequently sonicated for 30 min. Then, the preparation of process Al_2_O_3_/RGO involved mixing 5mmol glucose with 70ml of deionized water for 10 min, followed by the addition of 10mmol AlCl_3_•6H_2_O_3_ and 10mmol NaAlO_2_, and stirring for 30 min. Subsequently, the well-dispersed GO solution was added and the mixture was stirred for an additional 1 h. The stirred solution was then transferred to a 100ml stainless steel autoclave lined with Teflon and maintained at 150 °C for 24 h. Following the reaction, the sample was washed with deionized water and ethanol, and then dried at 60 °C for 24 h. The resulting Al_2_O_3_/RGO complex was crushed to produce Al_2_O_3_/RGO. Synthesis of Al_2_O_3_/RGO is shown in Fig. 2.

### Prepare of RGO, Al_2_O_3_/RGO and Al_2_O_3_/RGO/Al6061 composite

The Al6061 powder was introduced to a high-speed mixer and blended with varying ratios (0.1, 0.3, and 0.5 wt%) of Al_2_O_3_/RGO for one hour to achieve a preliminary mixture. Following the blending process, the powder was transferred to a ball mill tank and subjected to planetary ball milling at 200 rpm for 10 h, alternating between 20 min of forward rotation and 20 min of reverse rotation. The milling was conducted with a ball to material ratio of 5:1, consisting of 20% of 10 mm balls, 60% of 8 mm balls, and 20% of 5 mm balls.

To create the preforms, the resulting mixture from ball milling was loaded into a 40 mm diameter extrusion barrel die and compressed at 300 MPa for 5 min. Once the extrusion die with a 16:1 extrusion ratio was mounted onto the extrusion barrel and securely fastened, the entire assembly, including the preforms, was transferred to a heating furnace. The furnace was preheated to 500 °C at a rate of 200 °C/h. After holding at 500 °C for 50 min, the process of hot extrusion commenced, yielding a composite aluminum alloy bar with a diameter of 10 mm.

**Figure 3:**
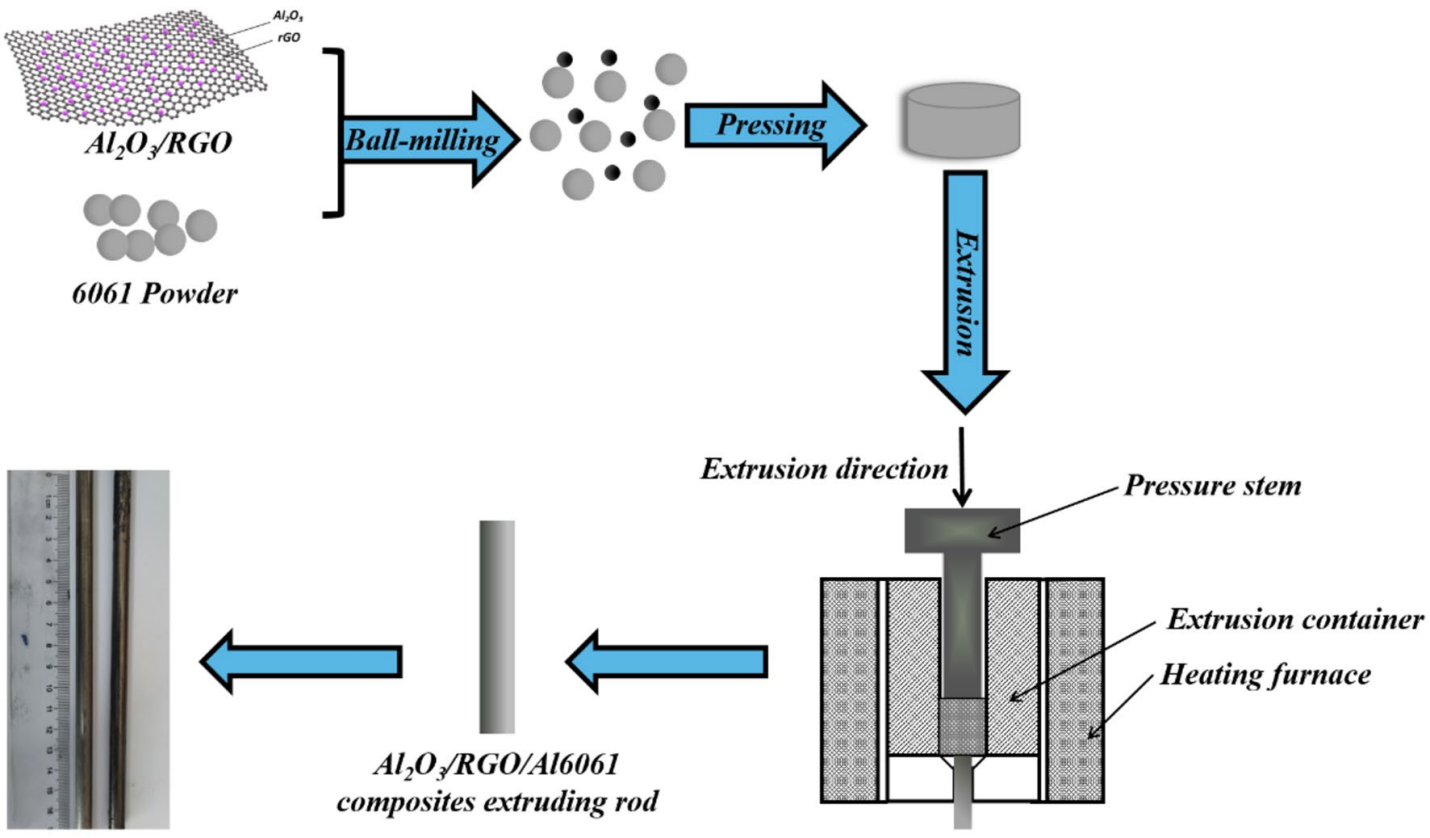
Schematic diagram of the preparation process of Al_2_O_3_/RGO/Al6061 composite.

For comparison, pure Al6061 samples without the incorporation of Al_2_O_3_/RGO were prepared using the same methodology. Figure 3 provides a schematic representation of the preparation process.

### Characterization method

Density measurements were carried out on Al6061 and Al MMCs specimens using Archimedes' principle, and the density of the composite was calculated using the Eq. (1).

1 $$\rho_{m} = \frac{m}{{m - m_{1} }}\rho_{w}$$

where $m$ is the mass of the composite sample in air, $m_{1}$ is the mass of the same composite sample in distilled water and $\rho_{w}$ is the density of distilled water. The density of distilled water at 20 °C is $998\,\text{kg/m}^{3}$.

The microstructure of the obtained Al MMCs was observed using scanning electron microscopy (SEM) with the GeminiSEM 500. The phase composition was determined through X-ray diffraction (XRD) analysis using an energy dispersive X-ray spectrometer (EDS) and a Mini Flex 300/600. The specimens were exposed to Cu Kα radiation (0.15418 nm) with a scanning speed of 2°/min, and the 2θ scans were conducted in the range of 20° to 90°. Sheet samples of the alloy cross sections were manually crushed into thin foils less than 100um thick and punched into 3mm diameter plates with a puncher. The plates were electrolytically flattened with a twin-jet electropolishing machine before being inspected using a JEM-2100F transmission electron microscope (TEM) at 200 kV. The electrolyte was a solution of 10% perchloric acid in ethanol.

The hardness of extruded bars was evaluated using a Wilson|VH1102-01-0087 hardness tester in accordance with ISO 6507. The mechanical properties of the Al MMCs were assessed following ASTM E8M and ISO 6892-1 standards. Tensile specimens with a diameter of Φ5 were machined from the extruded bars and tested at room temperature using an E45.305 electronic universal testing machine (300 kN) at a constant rate of 0.1 mm/min. To ensure the statistical significance of the results, at least three samples were tested for each condition. After the tensile tests, the fracture surfaces and regions near the fractures of the specimens were examined using SEM.

## Results and discussion

**Figure 4:**
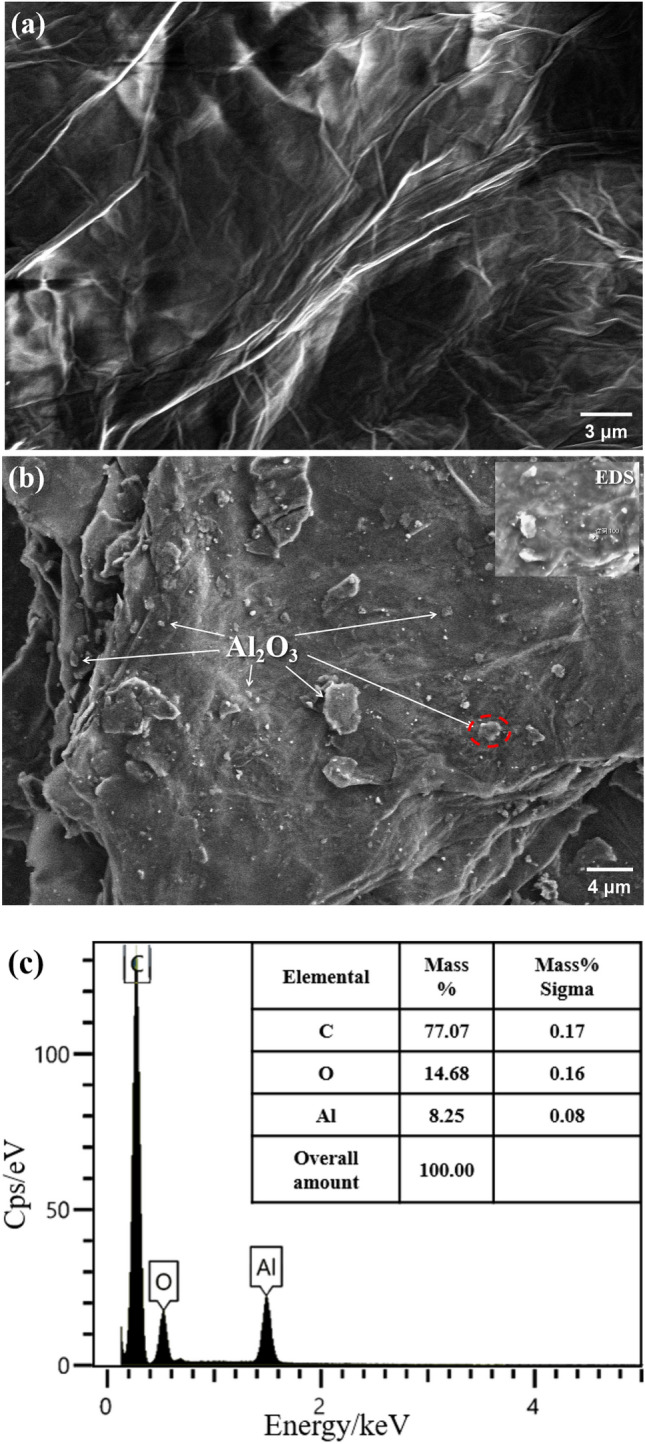
SEM images of GO (**a**), Al_2_O_3_/RGO (**b**) and EDS of Al_2_O_3_/RGO (**c**).

The morphology of GO is illustrated in Fig. 4a. GO exhibits a two-dimensional layered structure with undulating and wrinkled features, which demonstrate the high specific surface area of GO. Figure 4b shows the morphology of Al_2_O_3_/RGO, which many small particles adhered to layered RGO. Based on the EDS results (Fig. 4c at red circle area in Fig. 4b), additionally, it can be confirmed that the particles on the surface of RGO were Al_2_O_3_, indicating that Al_2_O_3_ has been successfully loaded onto RGO. The Al_2_O_3_/RGO composite has an average diameter of ranging from 400 nm to 4 um.

**Figure 5:**
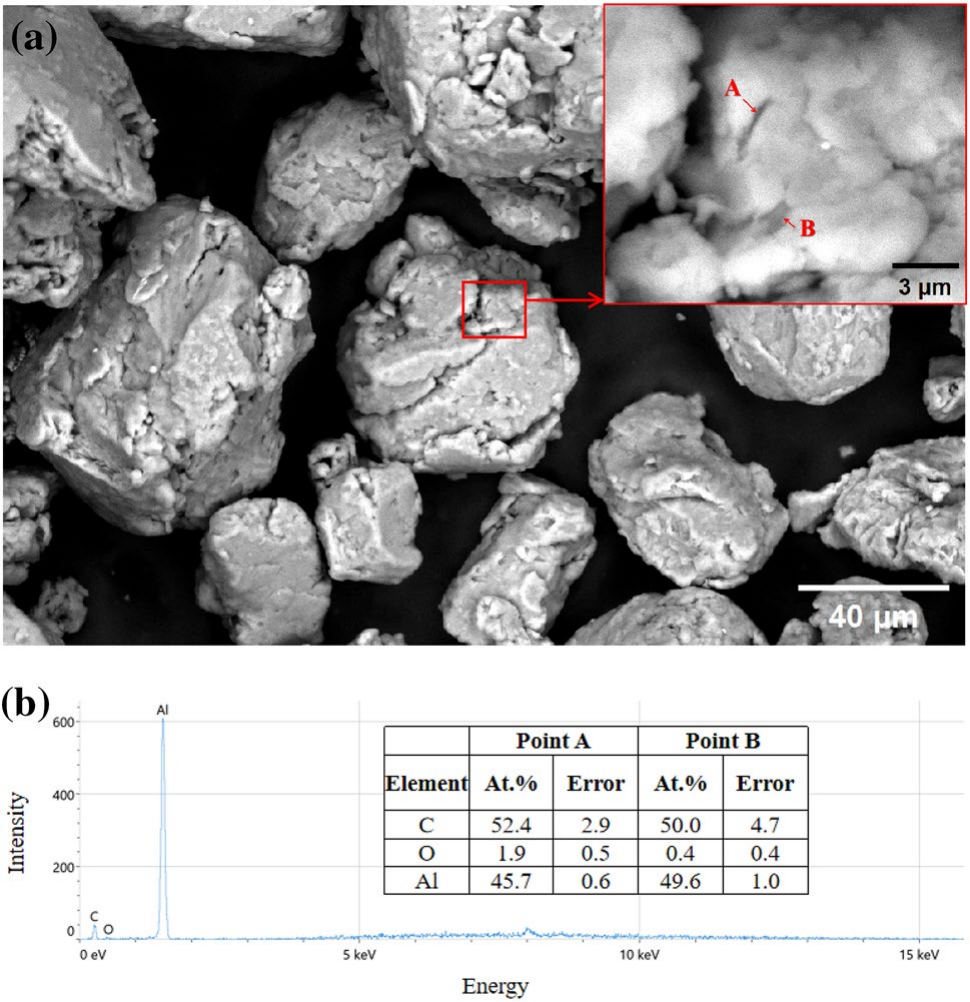
The typical SEM image of Al6061 powder with 0.1 wt.% Al_2_O_3_/RGO after ball mill (**a**) and EDS point scan results of (**a**) at A and B area (**b**).

As shown in Fig. 5a, it can be found that the addition of reinforcements has caused modifications in the morphology of the matrix composite. The initial spherical Al6061 powder has evolved into a polygonal shape with fractures spread throughout. This might be related to the flattening and cold welding of spherical particles during the ball milling procedure. As depicted in Fig. 5b, the EDS point scan findings of Zone A and B in Fig. 5a (indicated within a red box) show that the black strip are RGO, which are securely lodged in the aluminum alloy matrix by ball milling.

**Figure 6:**
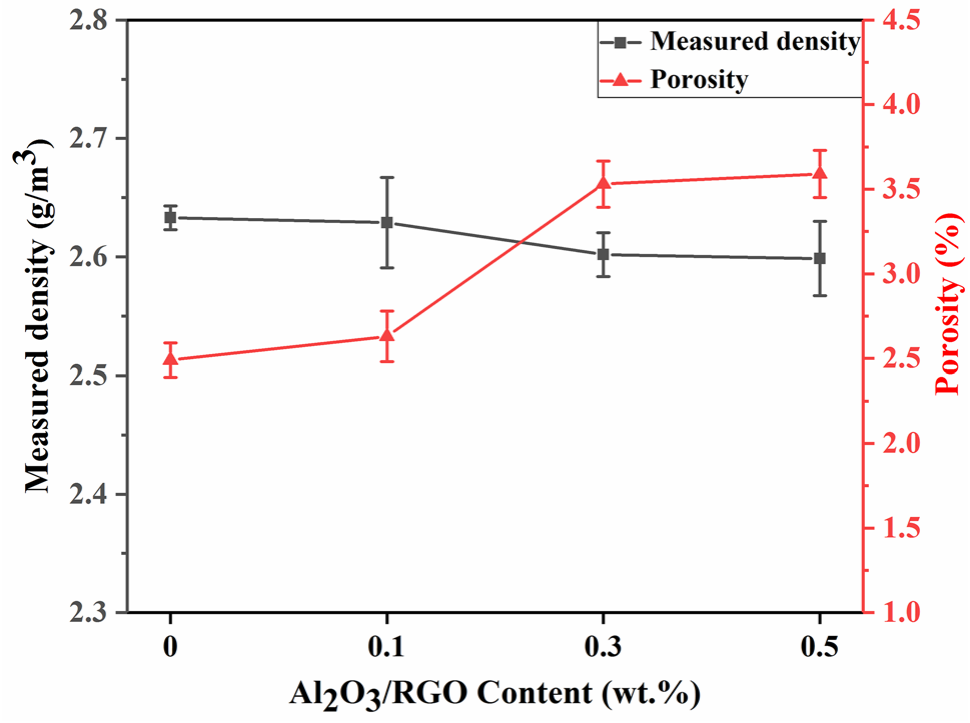
Measured density and porosity of Al MMCs with 0, 0.1, 0.3 and 0.5 wt% Al_2_O_3_/RGO.

Figure 6 illustrates the changes in density and porosity of Al6061 and Al MMCs. It is evident that as the Al_2_O_3_/RGO ratio increase, the density of Al MMCs decreases while the porosity increases, aligning with the theoretical calculations. The increase in porosity, which can be attributed to factors such as layered structure of RGO and a considerable difference in the linear expansion coefficients between graphene and the Al6061 matrix^23^.

**Figure 7:**
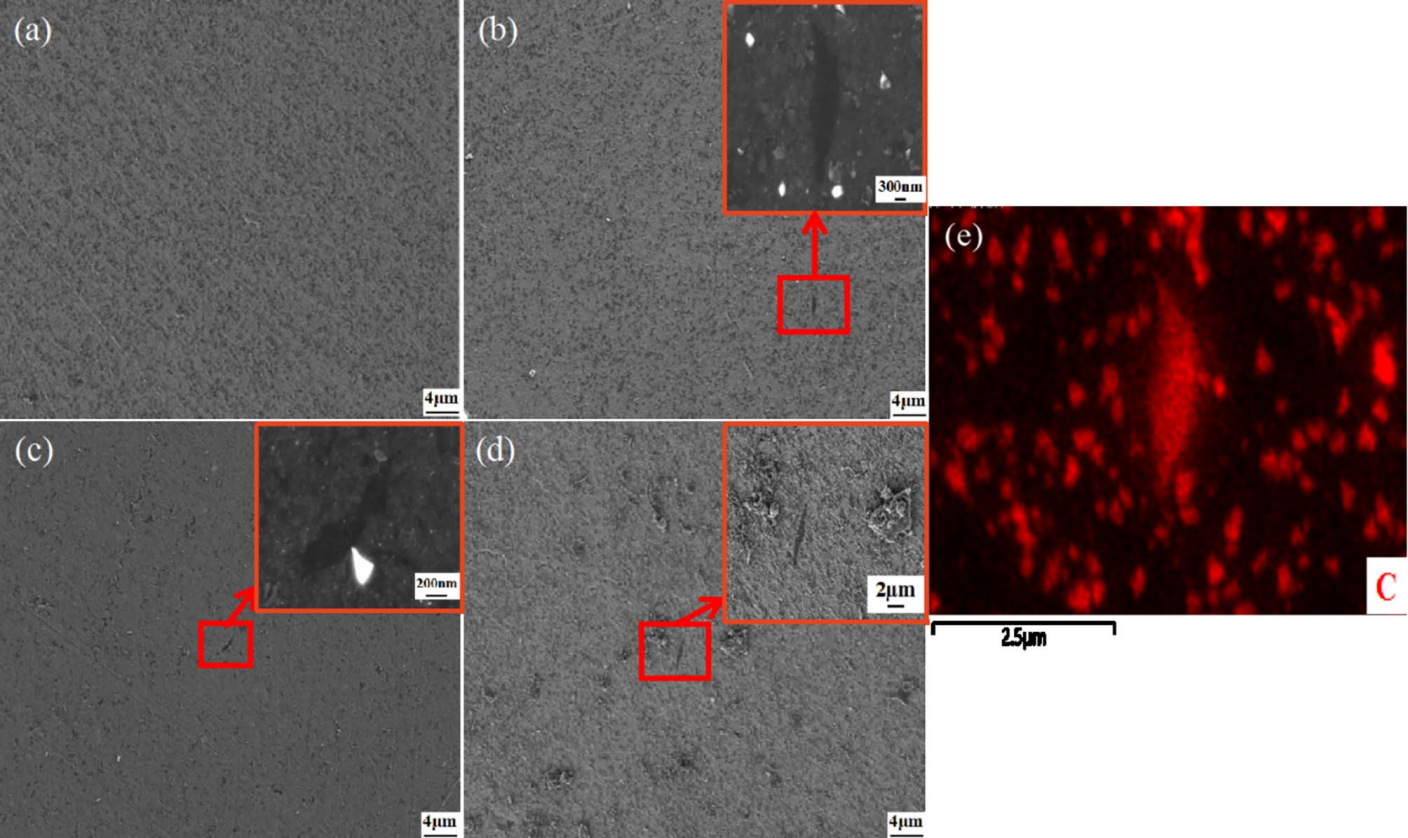
The typical SEM image of Al6061 (**a**), 0.1 wt% Al_2_O_3_/RGO/Al6061 (**b**), 0.3 wt% Al_2_O_3_/RGO/Al6061 (**c**), 0.5 wt% Al_2_O_3_/RGO/Al6061 (**d**) and Al_2_O_3_/RGO EDS spectrum (**e**) of (**b**).

Figure 7 displays the transverse SEM microstructure of Al MMCs with varying mass percentages of Al_2_O_3_/RGO. The microstructure of the four specimens, produced using the same process but with different mass fractions of Al_2_O_3_/RGO, exhibited general similarities, as depicted in Fig. 7a-d. Notably, no discernible metallurgical defects such as shrinkage, bubbles, or cracks were observed. Following the addition of Al_2_O_3_/RGO reinforcement, fine black strips emerged within the Al6061 matrix, and the morphology is consistent with Fig. 5b. In Fig. 7b (Al_2_O_3_/RGO: 0.1 wt%), the flaky Al_2_O_3_/RGO reinforcement is visible on the surface of the Al6061 composite (indicated within a red box), with its size ranging from and the elements in its region confirmed through EDS analysis, as shown in Fig. 7e.

**Figure 8:**
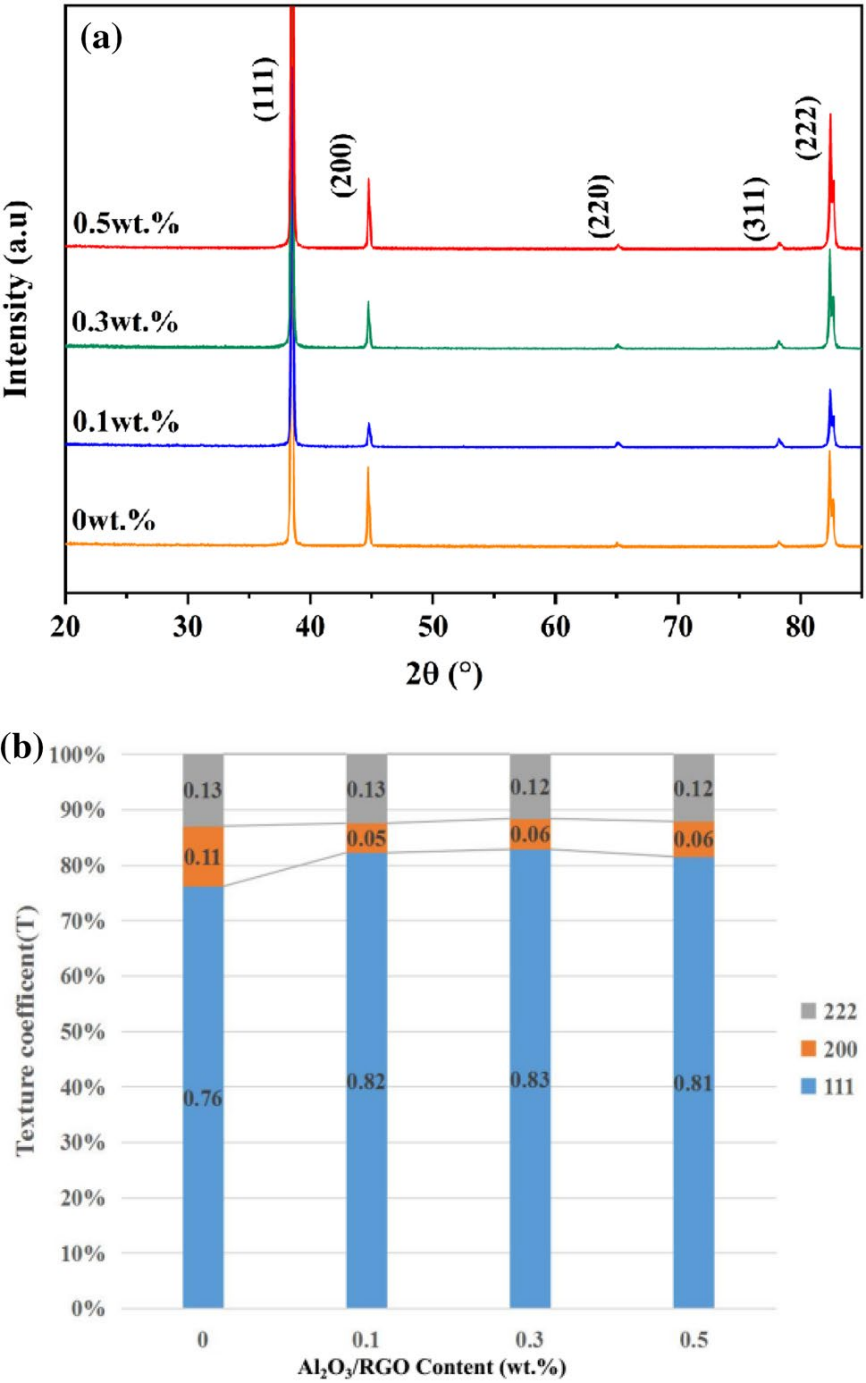
XRD pattern (**a**) and texture coefficients (**b**) of Al MMCs with 0, 0.1, 0.3 and 0.5 wt% Al_2_O_3_/RGO.

The XRD diffraction patterns of Al MMCs with varying Al_2_O_3_/RGO content after hot extrusion are depicted in Fig. 8. It is evident that the primary peaks correspond to the Al alloy, and the crystal structure remains unchanged following the addition of Al_2_O_3_/RGO reinforcement and the hot extrusion process. As per JCPDS (Joint Committee on Powder Diffraction Standards) card number 85-1327, the peaks at 38.3°, 44.6°, 65.1°, 78.2°, and 82.3° correspond to the crystallographic indices of (111), (200), (220), (311), and (222), respectively. Notably, peaks related to the RGO, Al_2_O_3_ and Al_4_C_3_ phase are absent in the XRD patterns due to their minimal presence. Unlike finds in other studies^29,30^, there was no brittle phase of Al_4_C_3_ at the end of the hot extrusion preparation. This is attributed to the RGO, which is coated in Al_2_O_3_ particles, exhibiting limited interaction with the Al6061 matrix, thereby hindering the formation of the Al_4_C_3_ brittle phase. The texture coefficient of the alloy was calculated using the equation (Eq. 2), where I is the intensity of diffraction peak, hkl denotes the (111), (200), or (222) orientation^33^.

2 $$\text{Texture coefficient}=\frac{I(hkl)}{I\left(111\right)+I\left(200\right)+I(220)}$$

The texture coefficients of the composites at various mass percentages are presented in Fig. 8b, revealing a reduction in the texture coefficient for the (200) orientation following the addition of the Al_2_O_3_/RGO compound. These texture modifications suggest that the grain orientation underwent transformation due to the presence of the Al_2_O_3_/RGO phase.

**Figure 9:**
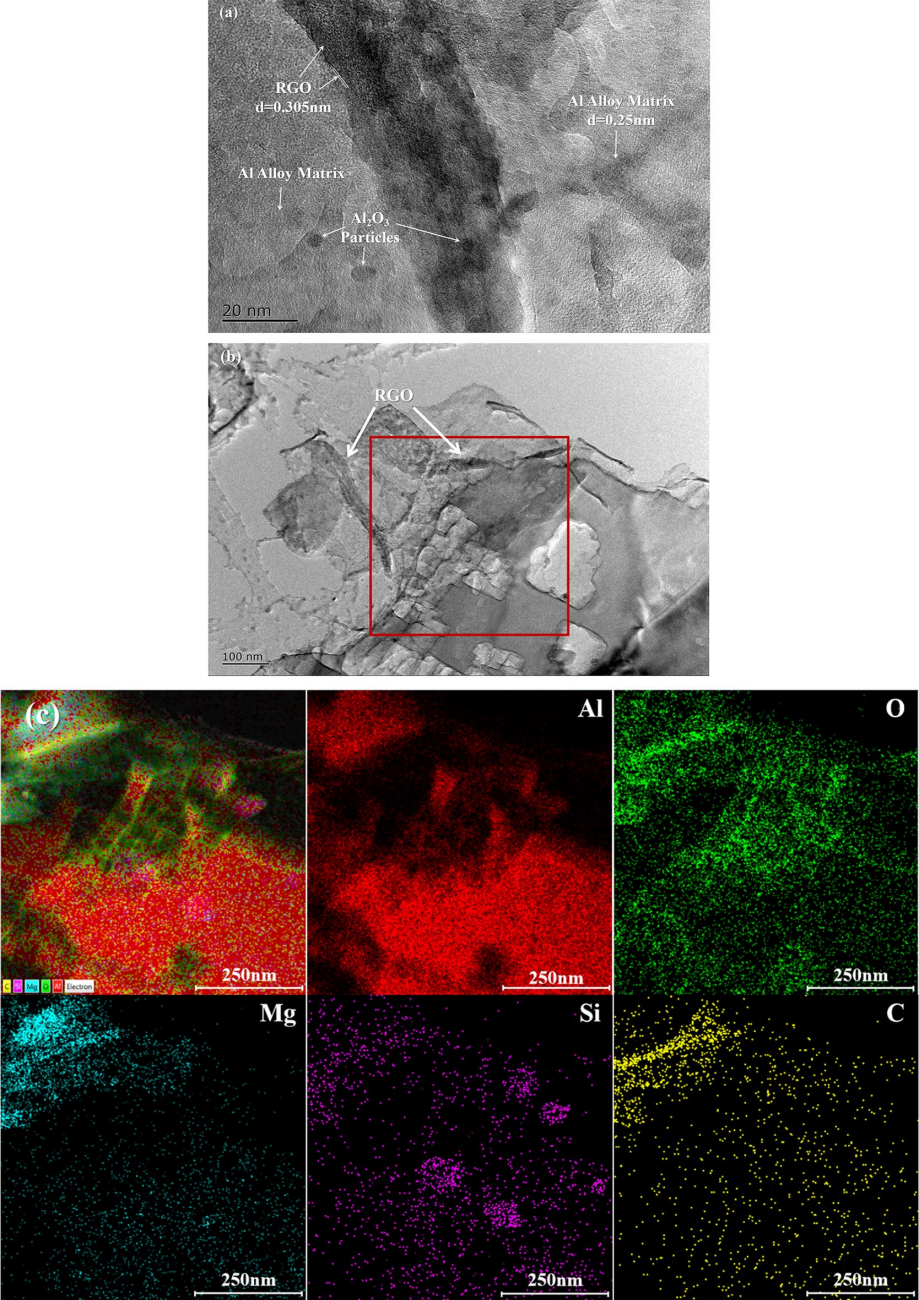
Bright-field TEM and EDS images of 0.1 wt% Al_2_O_3_/RGO/Al6061 composites: the high magnification image showing Al_2_O_3_/RGO fragments embedded in the composites (**a**), low-magnification images showing Al_2_O_3_/RGO in composites (**b**), the EDS mapping images of 0.1 wt% Al_2_O_3_/RGO/Al6061 composites in the red-framed area (**c**) of (**b**).

Typical bright-field TEM and EDS images of 0.1wt% Al_2_O_3_/RGO/Al6061 composites are shown in Fig. 9. In the high magnification image in Fig. 9a, large black stripes of RGO and small particles of Al_2_O_3_ are embedded in the grey Al6061 matrix. The Al_2_O_3_/RGO fragments and matrix are tightly bound together. The brittle Al_4_C_3_ phase was not found in the TEM image, in agreement with the XRD results. Figure 9b shows the low magnification TEM images of graphene sheets in the composites. And Fig. 9c is the EDS mapping images of 0.1wt% Al_2_O_3_/RGO/Al6061 composites in the red-framed area of Fig. 9b. The RGO was identified by C element distribution and O element enrichment. Additionally, an enrichment of Mg elements near the RGO is observed, while a Si rich phase is found in the Al matrix.

**Figure 10:**
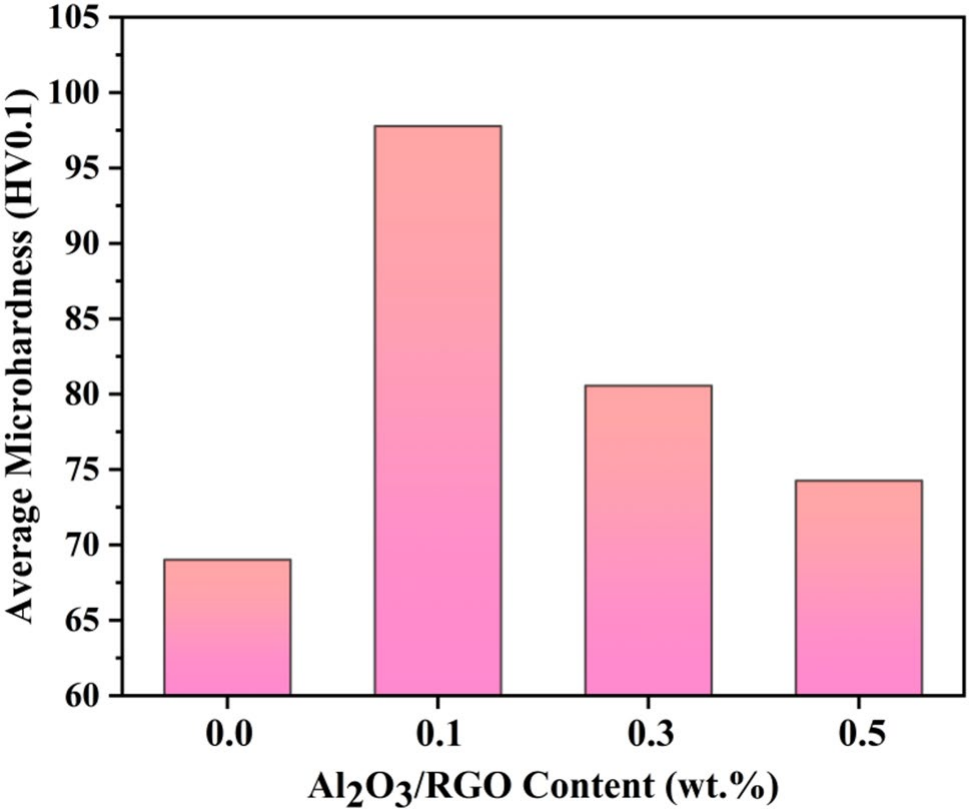
Average microhardness of Al MMCs with 0, 0.1, 0.3 and 0.5 wt% Al_2_O_3_/RGO.

Figure 10 depicts the average microhardness of Al MMCs. As the Al_2_O_3_/RGO content in the Al 6061 matrix rises, the microhardness values of the Al MMCs experience a substantial boost. Specifically, their hardness escalates from 69.02 to 97.78 Hv, marking a remarkable 41.6% increase. This clearly demonstrates the beneficial impact of Al_2_O_3_/RGO on Al6061.

**Figure 11:**
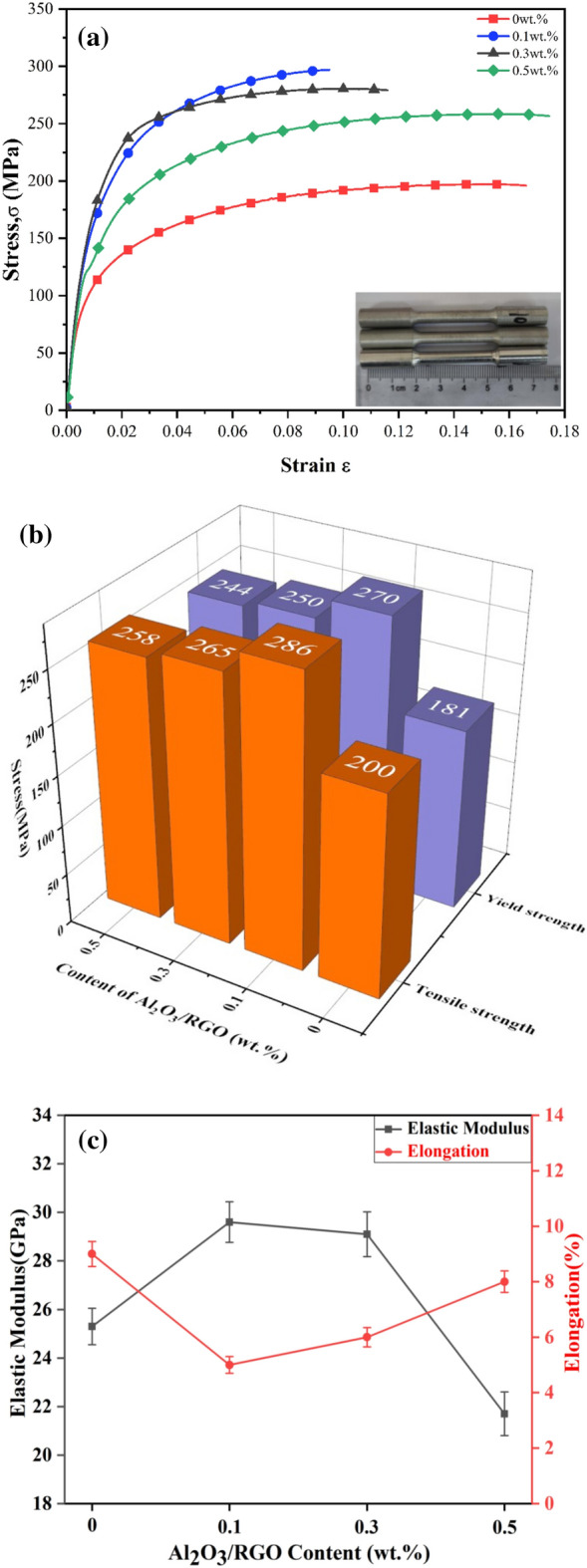
Representative tensile behavior of Al MMCs with 0, 0.1, 0.3 and 0.5 wt% Al_2_O_3_/RGO: True stress–strain curves of Al MMCs (**a**), tensile strength, yield strength (**b**), and modulus of elasticity, elongation (**c**).

Figure 11 demonstrate the mechanical properties of Al MMCs with varying Al_2_O_3_/RGO contents. Figure 11a showcases the true stress–strain curves of the Al MMCs. As depicted in Fig. 11b and c, the addition of Al_2_O_3_/RGO led to a slight decrease in plasticity but an increase in strength for the Al MMCs. The incorporation of different amounts of reinforcement significantly enhanced the yield strength and tensile strength of the composites compared to pure Al6061. The maximum improvement was observed at a content of 0.1 wt%. Specifically, the yield strength increased by 49%, rising from 181 to 270 MPa, while the tensile strength improved by 43%, increasing from 200 to 286 MPa. Additionally, the reinforcement had a considerable impact on the elastic modulus of the composites, initially increasing and then declining. These results unequivocally demonstrate the substantial enhancement of the mechanical characteristics of the composites through the inclusion of the Al_2_O_3_/RGO phase. Similar findings were also reported by Saravanan^34^.

**Figure 12:**
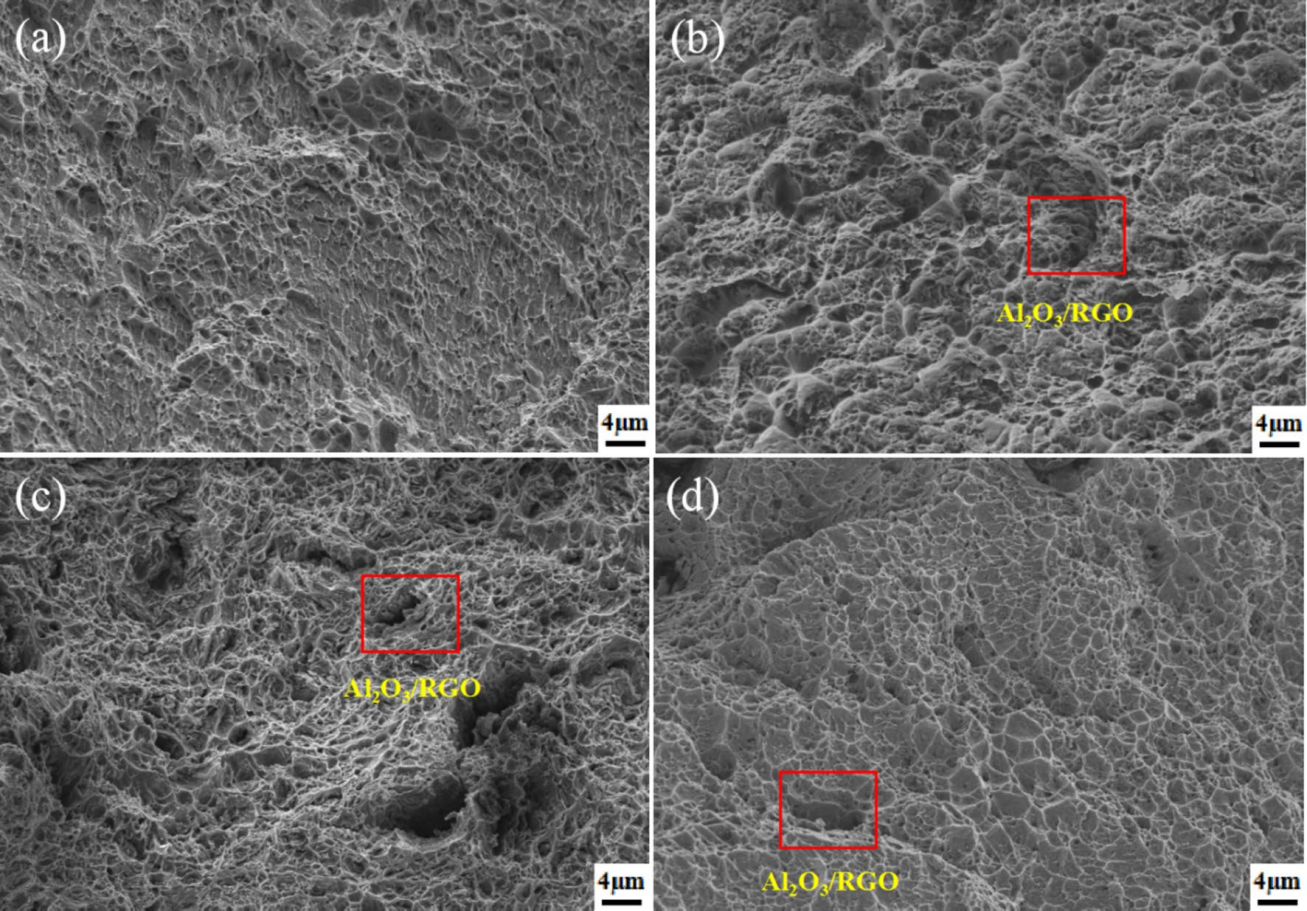
Fracture morphology of Al MMCs with 0 wt% (**a**), 0.1 wt% (**b**), 0.3 wt% (**c**) and 0.5 wt% (**d**).

Figure 12 illustrates the fracture surfaces of Al6061 with the Al_2_O_3_/RGO phase. Dimples and tearing ridges were observed in the alloys, regardless of the presence of the Al_2_O_3_/RGO phase (Fig. 12a). Initially, the size of the dimples decreased with increasing content of the enhancement phase, but then increased, aligning with the variation in tensile elongation of the Al MMCs investigated. In Fig. 12b–d, the Al_2_O_3_/RGO phase can be seen being pulled out from the matrix, forming strip-like voids. This observation clearly demonstrates the effective strengthening effect of the enhancement phase.

The strengthening mechanisms of second-phase reinforced metal matrix composites can be attributed to many sources, such as load transfer strengthening, grain refinement strengthening, coefficient of thermal expansion (CTE) strengthening, and Orowan strengthening. In the present investigation, the reinforcements are two-dimensional Al_2_O_3_-decorated RGO particles, thus necessitating the consideration of all four reinforcement mechanisms. Therefore, the multiple strengthening mechanisms operating in RGO and Al_2_O_3_ synergistic reinforced Al MMC can be expressed as Eq. (3):

3 $${\sigma }_{c}={\sigma }_{m}+{\sigma }_{LT}+{\sigma }_{GR}+{\sigma }_{CTE}+{\sigma }_{OR}$$

where ${\sigma }_{m}$ is the yield strength of Al6061 (180MPa, in this study), and ${\sigma }_{LT}$, ${\sigma }_{GR}$,${\sigma }_{CTE}$ and ${\sigma }_{OR}$ is the effect of load transfer strengthening, grain refinement strengthening, CTE strengthening, and Orowan strengthening, respectively.

In this study, the load transfer strengthening of RGO provide the important part of the total strengthening. The effectiveness of the load transfer can be quantified using the load transfer model specific to RGO as outlined in Eq. (4)^35^:

4 $${\sigma }_{LT}=\left(\frac{S}{4A}-1\right){V}_{RGO}{\sigma }_{m}$$

where S is the interfacial area between RGOs and Al6061 matrix, A refers to the cross-sectional area along the tensile direction. According to Fig. 9, the average size of RGO is $20\,\mu \text{m}\times 20\, \mu\text{m}\times 10\,\text{ nm}$, and the V_RGO_ is volume percentage of RGO, calculated from weight content of RGO. The load transfer strength for 0.1, 0.3 and 0.5 wt% Al_2_O_3_/RGOs are 27, 76 and 134 MPa, respectively. High interfacial transfer efficiency is contingent upon the presence of elevated shear stress at the interface^36^. The presence of Al_2_O_3_ on the surface of RGOs confines the RGO within the Al6061 matrix, tightly, thus increasing the critical shear stress value at the interface between the RGOs and the matrix.

Ball milling facilitates the incorporation of the Al_2_O_3_/RGO into the aluminum alloy powder, thereby enabling the suppression of grain growth during subsequent sintering and hot extrusion processes. According to the XRD results, the grain size of aluminum alloy composite materials can be calculated by the Williamson-Hall formula^14,37^. The yield strength of Al MMCs enhanced through grain refinement strengthening can be calculated by Hall–Petch relationship, as shown in Eq. (5).

5 $${\sigma }_{GR}=k{(d}_{c}^{-0.5}-{d}_{m}^{-0.5})$$

where k is a constant (0.08 MPa m for Al alloy^38^), d_c_ and d_m_ are the average grain size of Al_2_O_3_/RGO/Al6061 composite and pure Al6061, respectively. The strength improvements of grain refinement with 0.1, 0.3 and 0.5 wt% Al_2_O_3_/RGO are 48, 20 and 8MPa.

The residual stress induced by the mismatch in thermal expansion coefficients between the matrix and the particles may lead to the formation of dislocations around the particles, resulting in an increase in tensile strength. The thermal mismatch strengthening of the matrix can be quantified utilizing the subsequent Eq. (6) and (7)^39^.

6 $${\sigma }_{CTE}^{{Al}_{2}{O}_{3}}=a{G}_{Al}\sqrt{\frac{Bb{V}_{{Al}_{2}{O}_{3}}\Delta \alpha \Delta T}{{D}_{{Al}_{2}{O}_{3}}(1-{V}_{{Al}_{2}{O}_{3}})}}$$

7 $${\sigma }_{CTE}^{RGO}=a{G}_{Al}\sqrt{\frac{Bb{V}_{RGO}\Delta \alpha \Delta T}{{D}_{RGO}(1-{V}_{RGO})}}$$

where a is the geometric constant (0.83)^40^, G_Al_ is the shear modulus of Al (2.6 × 10^4^ MPa), B is a constant between 4 (RGO) and 12(Al_2_O_3_ particles)^41^, b is burgers vector (2.86 × 10^−10^ m)^42^ of Al, Δα is the difference in thermal expansion coefficients of the matrix and reinforcements (24 × 10^−6^ K^−1^,  − 6 × 10^−6^ K^−1^ and 7 × 10^−6^ K^−1^ for aluminum, RGO and Al_2_O_3_)^43^. ΔT is the difference between the test temperature (298 K) and the hot extrusion temperature (798 K), ${D}_{{Al}_{2}{O}_{3}}$ and ${D}_{RGO}$ is the diameter of the Al_2_O_3_ and RGO, ${V}_{{Al}_{2}{O}_{3}}$ is volume percentage of Al_2_O_3_. The CTE strengthening increment of RGO and Al_2_O_3_ with different volume fraction is given in Table 2.

**Table 2:** Calculated and experimental yield strength of strengthening mechanism of composites.

Content of Al_2_O_3_/RGO (wt%)	${\sigma }_{LT}$ (MPa)	${\sigma }_{GR}$ (MPa)	${\sigma }_{CTE}^{{Al}_{2}{O}_{3}}$ (MPa)	${\sigma }_{CTE}^{RGO}$ (MPa)	${\sigma }_{OR}$ (MPa)	${\sigma }_{c}$ (MPa)	Experimental yield strength (MPa)
0.1	27	48	3.3	0.65	6	265	270
0.3	76	20	5.5	1	8.5	291	250
0.5	134	8	7	1.43	10.6	341	244

Because of the small dimensions of Al_2_O_3_ particles adhered to RGO, the Orowan strengthening effect of Al_2_O_3_ particles can be anticipated by applying the Orowan strengthening model, as shown in Eq. (8)^36,44^. The Orowan strengthening increment of Al_2_O_3_ with different volume fraction is also given in Table 2.

8 $${\sigma }_{OR}=0.13\frac{{G}_{Al}b}{{D}_{{Al}_{2}{O}_{3}}\left[{\left(1/2{V}_{{Al}_{2}{O}_{3}}\right)}^{1/3}-1\right]}ln\left(\frac{{D}_{{Al}_{2}{O}_{3}}}{2b}\right)$$

The contributions of Al_2_O_3_/RGO reinforcement corresponding to the four strengthening mechanisms are shown in Table 2. The reinforcement effect of the composite material depends on the characteristics of the reinforcement. RGOs mainly play the role of load transfer, while the Al_2_O_3_ phases play the role of Orowan strengthening. The synergistic effect of Al_2_O_3_ and RGO improves interface bonding performance and enhances particle dislocation precipitation interaction, thereby enhance total composite material strength. It can be found the theoretically enhancement effect increases with the rise in the reinforcing phase content. However, a comparison with experimental findings reveals that when the reinforcing phase content surpasses 0.1 wt%, the calculated value become higher than the experimental value. The increase in particle volume fraction primarily leads the agglomeration of particles, reduction in the surface area of graphene interfacing with the matrix, and a decrease in interfacial bonding strength^45^. These factors collectively promote the initiation and propagation of cracks during tensile processes, consequently leading to a decline in the tensile strength of Al MMC with higher content of reinforcing phase. Thus, employing the suitable process techniques to control the size of the reinforcing phase and prevent their agglomeration^8,18^ is important to the synergistic reinforcement effect of Al_2_O_3_ and RGO on Al MMC.

**Figure 13:**
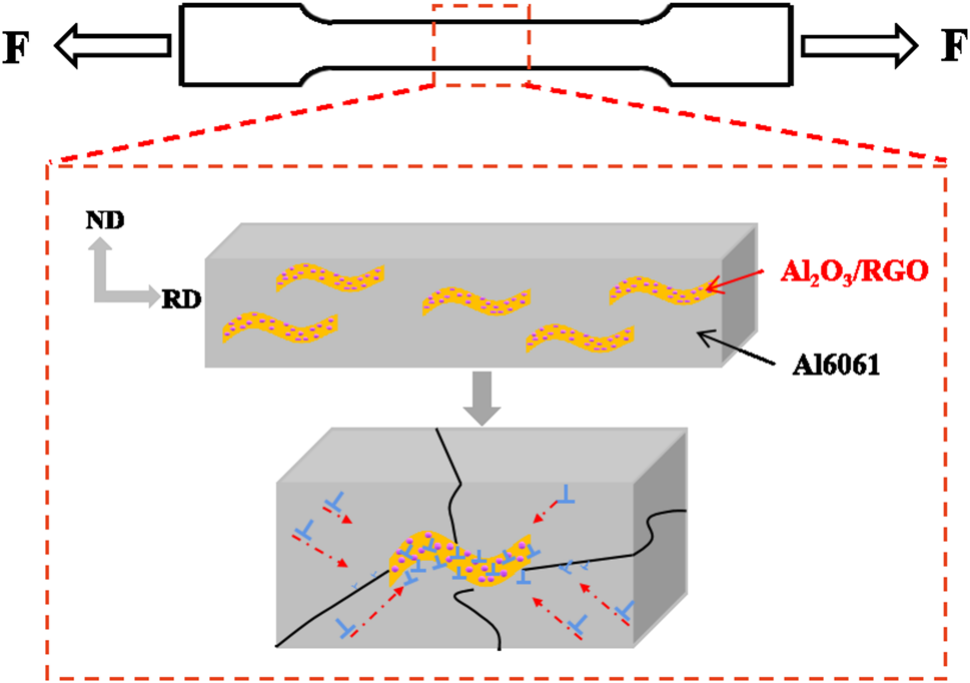
Schematic diagram of the deformation mechanism of the Al MMCs.

Figure 13 presents an analysis of the fracture mechanism of Al MMCs to reveal their behavior under tensile forces. After the process of hot extrusion, the distribution of Al_2_O_3_/RGO in Al MMC demonstrates a certain degree of directionality. Initially, the matrix undergoes plastic deformation, resulting in the formation of numerous microcracks. As the tensile force increases, these microcracks progressively expand inward alongside the matrix, accompanied by an increase in dislocation movement. However, when these dislocations encounter the obstacles presented by Al_2_O_3_/RGO particles, their extension is impeded. The strengthening occurs when the movement of dislocations is hindered. Additionally, Al_2_O_3_ particles were deposited onto RGO to increase its surface roughness and facilitate better interaction with the Al matrix. This could lead to the formation of an interlocking effect between the Al_2_O_3_/RGO reinforcement and the Al6061 matrix, which further strengthen the material^46^. Furthermore, due to the disparate thermal expansion coefficients of Al6061 and Al_2_O_3_/RGO nanoparticles, strain fields are created around the Al_2_O_3_/RGO during the cooling process following hot extrusion. These strain fields act as barriers to dislocation movement during stretching. Consequently, a higher load is required to transfer these dislocations around the strain field. The fracture morphology of the stretched specimen in Fig. 13 further supports and elucidates this phenomenon.

## Conclusion

In sum, Al6061 alloy matrix composites reinforced with different content of reduced graphene oxide loaded Al_2_O_3_ were successfully manufactured by high-energy ball milling and hot extrusion processes. The Al_2_O_3_/RGO is uniformly embedded in the Al6061 matrix and strongly bonded to the matrix. Because Al_2_O_3_ covered the RGO surface to inhibite the reaction between the RGO and the Al alloy matrix, there was no formation of the brittle Al_4_C_3_ phase. The alloy’s yield strength and tensile strength exhibited a sharp increase followed by a slow decrease with increasing Al_2_O_3_/RGO content. The peak values for yield and tensile strengths, reaching 270 MPa and 286 MPa, were attained with a 0.1 wt% reinforcement. These values represented a 49% increase in yield strength and a 43% increase in tensile strength compared to the pure Al6061 alloy. The Al6061 with Al_2_O_3_/RGO exhibited comprehensive and excellent mechanical properties due to the synergistic effect of RGO and Al_2_O_3_.

## Data Availability

All data, models generated or used during the study are available from the corresponding author by request.

## List of symbols

Al MMC: Aluminum metal matrix composite
RGO: Reduced graphene oxide
Al_2_O_3_/RGO: Reduced graphene oxide loaded Al_2_O_3_
XRD: X-ray diffraction
SEM: Scanning electron microscopy
EDS: Energy-dispersive X-ray spectroscopy
TEM: Transmission electron microscope
GO: Graphene oxide
JCPDS: Joint committee on powder diffraction standards
CTE: Coefficient of thermal expansion
wt%: Weight percentage
$m$: The mass of the composite sample in air
${m}_{1}$: The mass of the same composite sample in distilled water
${\rho }_{m}$: The density of the composite
${\rho }_{w}$: The density of distilled water
${\sigma }_{c}$: The yield strength of Aluminum Metal Matrix Composite
${\sigma }_{m}$: The yield strength of Al6061 alloy
${\sigma }_{LT}$: The effect of load transfer strengthening
${\sigma }_{GR}$: The effect of grain refinement strengthening
${\sigma }_{CTE}$: The effect of coefficient of thermal expansion strengthening
${\sigma }_{OR}$: The effect of Orowan strengthening
$S$: The interfacial area between RGOs and Al6061 matrix
$A$: The cross-sectional area along the tensile direction
${V}_{RGO}$: Volume percentage of RGO
${V}_{{Al}_{2}{O}_{3}}$: Volume percentage of Al_2_O_3_
$k$: The Hall Page coefficient
${d}_{c}$: The average grain size of Al_2_O_3_/RGO/Al6061 composite
${d}_{m}$: The average grain size of pure Al6061
${\sigma }_{CTE}^{{Al}_{2}{O}_{3}}$: The effect of coefficient of thermal expansion strengthening between Al_2_O_3_ and matrix
${\sigma }_{CTE}^{RGO}$: The effect of coefficient of thermal expansion strengthening between Al_2_O_3_ and RGO
$a$: The geometric constant
${G}_{Al}$: The shear modulus of Al
$B$: A constant between 4 (RGO) and 12 (Al_2_O_3_ particles)
$b$: The burgers vector
$\Delta \alpha$: The difference in thermal expansion coefficients of the matrix and reinforcements
$\Delta T$: The difference between the test temperature (298 K) and the hot extrusion temperature (798 K)
${D}_{{Al}_{2}{O}_{3}}$: The diameter of the Al_2_O_3_
${D}_{RGO}$: The diameter of RGO
